# The influence of mild hypothermia on reversal of rocuronium-induced deep neuromuscular block with sugammadex

**DOI:** 10.1186/1471-2253-15-7

**Published:** 2015-01-21

**Authors:** Hee Jong Lee, Kyo Sang Kim, Ji Seon Jeong, Kyu Nam Kim, Byeong Chan Lee

**Affiliations:** Department of Anesthesiology and Pain Medicine, Hanyang University Hospital, #17 Haengdang dong, Sungdong gu, Seoul, 133-792 Korea; Department of Anesthesiology and Pain Medicine, Samsung Seoul Hospital, Seoul, Korea

**Keywords:** Hypothermia, Neuromuscular blockade Rocuronium, Sugammadex

## Abstract

**Background:**

Mild hypothermia may be frequently induced due to cool environments in the operating room. The study analyzed patient recovery time and response to sugammadex after a prolonged rocuronium-induced deep neuromuscular block (NMB) during mild hypothermia.

**Methods:**

Sixty patients were randomly (1:1) allocated to the mild hypothermia and normothermia groups, defined as having core temperatures between 34.5 - 35°C and 36.5 - 37°C, respectively. Patients received 0.6 mg/kg of rocuronium, followed by 7 – 10 μg/kg/min to maintain a deep NMB [post-tetanic count (PTC) 1–2]. After surgery, the deep NMB was reversed with sugammadex 4.0 mg/kg. The primary end-point was the time until the train-of-four (TOF) ratio was 0.9.

**Results:**

The appropriate neuromuscular function (TOF ratio ≥ 0.9) was restored after sugammadex was administered, even after hypothermia. The length of recovery in the hypothermia patients [mean (SD), 171.1 (62.1) seconds (s)] was significantly slower compared with the normothermia patients [124.9 (59.2) s] (*p* = 0.005). There were no adverse effects from sugammadex.

**Conclusions:**

Sugammadex safely and securely reversed deep rocuronium-induced NMB during mild hypothermia. An additional 46 s was required for recovery from a deep NMB in hypothermia patients. Based on the results, we think this prolonged recovery time is clinically acceptable.

**Trial registration:**

ClinicalTrials.gov Identifier: NCT01965067.

## Background

Inadvertent perioperative hypothermia (<36°C) develops rapidly in the hour immediately after induction of general anesthesia, when exposed to a typical cool operating room environment [1]. Core temperature can be reduced by 1.6 degrees Celsius (°C) within the first hour and by 2.8°C during 3 hours in a 22°C air-conditioned environment [2]. Perioperative hypothermia has been associated with adverse patient outcomes, the most significant of which is an increased rate of myocardial ischemia, coagulopathies, postoperative shivering and wound infection [3–5]. The duration of vecuronium blockade is prolonged for twice the amount of time when the core temperature is decreased to 34.5°C [6]. So it is important to confirm the effect of core temperature to the reversal of rocuronium-induced neuromuscular block.

Sugammadex, a modified γ-cyclodextrin that encapsulates steroidal neuromuscular block (NMB) agents (such as rocuronium and vecuronium) in a highly selective fashion, has been shown to be reliable and complete, and can rapidly reverse moderate or deep NMB [7]. A deep NMB state [post-tetanic count (PTC) 1–2] was maintained until the closure or removal of the last device during laparoscopic surgery, and which can improve surgical conditions compared with moderate NMB [8]. The rapid and reliable antagonistic action of competitive NMB agents after surgery can enhance the safety and efficacy of the patients [9]. However, the reversal effect of sugammadex has not been investigated in mild hypothermia.

The primary objective of this study was to determine the reversibility of deep rocuronium-induced NMB for the PTC 1–2 steady block with sugammadex during mild hypothermia with core temperatures between 34.5°C and 35°C or normal thermal conditions.

## Methods

### Study subjects and study design

This randomized, parallel-group, safety-assessor-blinded phase IV study was approved from the Hospital Ethics Committee (IRB File No.: HYUH 2013-08-029-005), was conducted in accordance with principles of Good Clinical Research Practice, and written informed consent was obtained from the patients. The current study was registered at clinical trials.gov under the identification number NCT01965067 (Merck protocol no. MK8616-099).

Sixty patients of both sexes, ASA physical status I - II, aged between 21–64 years, that were undergoing elective abdominal surgery under general anesthesia were randomly assigned to either the hypothermia group (n = 30) (mild hypothermia with core temperatures between 34.5°C and 35°C) or the control group (n = 30) (normal thermal condition with core temperatures between 36.5°C and 37°C) using a computer-generated program. Patients were excluded if they were expected to have a difficult airway, suffered from neuromuscular, hepatic or renal diseases and undertaken the surgery on cardiopulmonary bypass; patients that were using any medication that might interact with muscle relaxants were also excluded in addition to pregnant women, and patients whose body mass index was < 18.5 kg/m^2^ or > 25 kg/m^2^.

### Study procedures

The patients were monitored with electrocardiography, non-invasive arterial pressure measurement, and pulse oximetry. Hypnotic depth was evaluated using a bispectral index (BIS) XP monitor (Model A 2000, Aspect Medical Systems, Newton, MA, USA). Anesthesia was started using propofol 2 – 2.5 mg/kg and remifentanil 0.5 μg.kg^−1^.min^−1^, and maintained with sevoflurane 1.1 – 1.6% end-tidal and remifentanil 0.1 – 0.3 μg.kg^−1^.min^−1^ to maintain a BIS monitor between 40 and 50 throughout surgery. Core body temperature was continuously observed by a thermocouple placed in the distal esophagus (DeRoyal®, DeRoyal Industries Inc., Powell, TN, USA). Core temperature was manipulated in core temperature ranges: 36.5°C and 37°C in the control group using forced-air warming (Bair Hugger Model 505, Arizant Healthcare, Eden Prairie, MN, USA), and 34.5°C and 35°C in the hypothermia group in a 22°C environment by surface cooling and the air conditioner fan. The skin temperature of the monitored arm was maintained at greater than 32°C with fixed temperature sensor at the distal end of the forearm in both groups.

Neuromuscular monitoring was performed with acceleromyography using the TOF-Watch SX® (Merck Sharp & Dohme Corp., Glostrup, Denmark) at the adductor pollicis. Two pediatric electrodes (Cleartrode^TM^, ConMed®, Utica, NY, USA) were placed over the ulnar nerve near the wrist. A 5-s 50-Hz tetanic stimulus was applied using the automated CAL2 mode to calibrate the TOF-Watch SX®, and then a supramaximal current was obtained after the initial single twitch calibration [10]. A bolus dose of rocuronium 0.6 mg/kg was injected and tracheal intubation was performed after a maximum NMB was achieved. Intermittent positive pressure ventilation was adjusted to maintain end-tidal carbon dioxide level between 30 and 35 mmHg. The ulnar nerve at the wrist was stimulated supramaximally with a TOF mode every 15 seconds (s). The PTC stimulation was initially performed 10 minutes (min) after obtaining complete NMB, and repeated manually every 6 min to monitor deep NMB throughout the study [11]. Rocuronium (7 – 10 μg.kg^−1^.min^−1^) was continuously infused to adjust to PTC 1 – 2 from 30 min after the initial dose of rocuronium [12]. After the surgical dressing, PTC 1 – 2 was confirmed on the TOF-Watch SX® reading, and then sugammadex 4 mg/kg was injected.

The primary objective was to evaluate recovery time and response to sugammadex after a prolonged rocuronium-induced deep NMB during mild hypothermia between 34.5°C and 35°C. Full recovery from the NMB was conducted during the administration of sevoflurane and remifentanil. The time to recovery of a train-of-four (TOF) ratio of 0.9 was evaluated and normalized by the baseline TOF ratio to improve the accuracy of NMB data [13]. Blood pressure, heart rate and BIS were measured at pre-reversal, post-reversal, recovery and post-anesthetic visits. The incidence of residual neuromuscular blockade, post-operative nausea and vomiting (PONV) and adverse events during mild hypothermia and the normal thermal condition were estimated for up to 7 days after administration of sugammadex by the assessor blinded to the study groups. Extubation was carried out after consciousness and regular respiration were confirmed. All neuromuscular results were stored in the computer and monitored until the end of the study. Risk factors for PONV were also assessed at baseline and used to assess the likelihood that any occurrences of PONV were associated with study therapy [14].

### Statistical analysis

For calculations, we used the statistical software package SPSS version 17.0 (SPSS Inc, Chicago, IL, USA). The sample size was calculated from the previous results of the averaged recovery time [mean (SD): 102 (42) seconds (s)] from deep rocuronium-induced NMB to a TOF ratio of 0.9 after sugammadex 4 mg/kg [15]. We considered that time would be prolonged by more than 50%, thus to 156 s (SD 66), to be clinically significant in patients during mild hypothermia. A sample size of 26 patients per group were needed for a power of 80% at a significance level of 5% (two-sided). We enrolled 30 patients per group to account for a 10% discontinuation rate. The results were presented as the mean values (SD or range). Categorical data was compared between groups using chi-squared analysis. Between-group comparisons were made using unpaired *t*-tests. A repeated measures ANOVA was used for the changes of blood pressure and heart rate. A *p*-value less than 0.05 was considered statistically significant.

## Results

**Figure 1:**
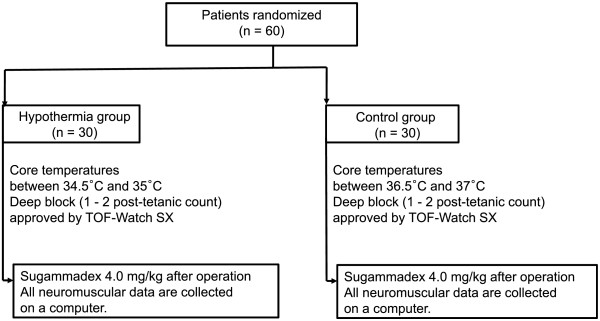
**Flow diagram of study participants in each group.**

Flow diagram of study participants in each group is shown in Figure 1. Sixty patients were enrolled in this study, and neuromuscular monitoring was successfully conducted. The two groups were similar in their demographic profile, anesthetic time, BIS, end-tidal concentration of sevoflurane, total dose of rocuronium, type of surgery, location of abdominal surgery, and core temperature (Table 1). The target core temperature in either hypothermia patients [34.8 (0.1)°C] or normothermia patients [36.7 (0.1)°C] was well maintained at the injection of sugammadex. In our study, the core temperature was steadily dropped during the course of the surgery in the hypothermia patients, and no rapid changes were observed.

**Table 1:** **Patient characteristics, duration of anesthesia, BIS, sevoflurane, rocuronium and core temperature**

	Hypothermia (n = 30)	Control (n = 30)	***p***
Male/Female (n)	13/17	13 / 17	1
Age, years, mean (range)	48 (26 – 64)	47 (21 – 61)	0.936
Weight, kg, mean (SD)	59.7 (10.1)	59.9 (7.0)	0.906
Height, cm, mean (SD)	162.6 (8.0)	163.2 (7.2)	0.788
ASA I/II (n)	19/11	21/9	0.622
Anesthetic time, hour, mean (SD)	3.1 (1.2)	2.7 (1.1)	0.221
BIS, mean (SD)	46.6 (9.5)	50.8 (8.0)	0.066
Sevoflurane, end-tidal volume %, mean (SD)	1.34 (0.1)	1.33 (0.1)	0.688
Rocuronium, mg, mean (SD)	113.2 (31.1)	115.8 (40.7)	0.787
Core temperature, °C, mean (SD)	34.8 (0.1)	36.7 (0.1)	< 0.001

*ASA*: American Society of Anesthesiologists; *BIS*: bispectral index; *SD*: standard deviation.

The mean recovery time to TOF ratio of 0.9 after sugammadex 4.0 mg/kg was 171.1 (62.1) s in the hypothermia group compared with 124.9 (59.2) s in the normothermia group (*p* = 0.005) (Table 2). For most patients (83%; 25/30 patients) in the normothermia group, the recovery time to the TOF ratio of 0.9 was less than 180 s. By comparison, in the hypothermia group, 60% of patients (18/30 patients) had recovered to a TOF ratio of 0.9 within 180 s.

**Table 2:** **Time from administration of sugammadex to recovery of TOF ratio to 0.7, 0.8, and 0.9**

	Hypothermia (n = 30)	Control (n = 30)	***p***
Time to TOF ratio 0.7, seconds, mean (SD) (range)	110.4 (44.6) (31–200)	81.9 (37.8) (29–163)	0.01
Time to TOF ratio 0.8, seconds, mean (SD) (range)	129.6 (49.8) (46–230)	93.9 (45.3) (29–193)	0.005
Time to TOF ratio 0.9, seconds, mean (SD) (range)	171.1 (62.1) (61–305)	124.9 (59.2) (46–298)	0.005

*TOF*: train-of-four; *SD*: standard deviation.

**Figure 2:**
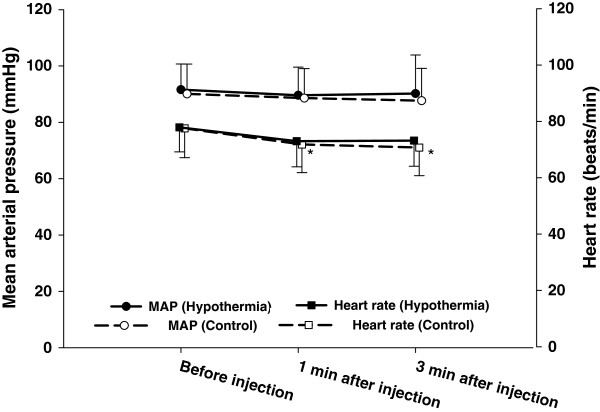
**Mean values (SD) for mean arterial pressure and heart rate for 3 minutes (min) in patients receiving sugammadex in deep rocuronium-induced neuromuscular block.** *p < 0.001 compared with values before sugammadex was injected.

The mean arterial pressure during the 180 s period following administration of sugammadex was not changed, but the heart rate during the same period decreased significantly after sugammadex was administered in both groups (*p* < 0.001) (Figure 2). Shivering was observed in four patients in the hypothermia group, which could be easily controlled by adjusting and administering meperidine 25 mg intravenously. PONV and any other adverse effects following administration of sugammadex were not reported in either group.

## Discussion

Our study confirmed that the recovery time to the TOF ratio of 0.9 after sugammadex administration in deep NMB was prolonged by 46 s during mild hypothermia (171.1 s) compared with the normal thermal condition (124.9 s).

Recovery at doses of sugammadex 4 mg/kg in a deep block at PTC 1–2 occurred within 120 s in younger adults [16]. Similar recovery time (162 s) after sugammadex at PTC 1–2 was reported [17]. Our study also demonstrated that 4 mg/kg sugammadex reversed patients in the control group from a rocuronium-induced NMB within 124.9 (59.2) s. However, sugammadex reversed the state within 171.1 (62.1) s from a rocuronium-induced deep NMB in hypothermia patients. The mechanism by which hypothermia prolongs the reversal from deep NMB with sugammadex remains controversial. The duration of action and rocuronium recovery were prolonged by moderate hypothermia (nasopharyngeal temperature: 30.4°C) due to reduced plasma clearance [18]. The adequate reversal of vecuronium block by neostigmine can be delayed during hypothermia (34.5°C) (more than 30 min) due to the prolonged duration of vecuronium and a decreased efficacy of neostigmine [6]. However, we found that, even in hypothermia, reversal with sugammadex was complete although hypothermia patients required additional 46 s to recover compared with the normothermia patients.

Studies have reported that cardiac output declined markedly in response to hypothermia [19, 20]. The onset of action of sugammadex may be affected by the cardiac output and muscle blood flow. The reduction of cardiac output is linearly associated with hypothermia, which is associated with decreased regional blood flow distribution compared with that of normothermia [19]. A lower regional blood flow implies that the delivery of the drug is reduced, which leads to a slower transfer rate of sugammadex and a slower reduction of free (unbound) rocuronium in the plasma. Although we could not measure the the pharmacokinetics of rocuronium and sugammadex, we suspect that this effect may reduce the speed of the recovery from NMB by sugammadex during hypothermia.

Good and optimal surgical conditions (99%) during deep NMB (good 32% and optimal 67%) were achieved with a higher frequency than during moderate NMB (82%) (good 48% and optimal 34%) in retroperitoneal laparoscopies [8]. In the present study, a PTC 1–2 in deep NMB is maintained during anesthesia with continuous infusion of rocuronium, which can achieve total diaphragmatic paralysis in response to tracheal suction [21]. We suspect that the ability to provide deep NMB throughout the procedure may enable improved surgical access and an enhanced visual field.

In adults, the infusion rate of rocuronium to maintain 10% of first twitch of TOF was 0.3 - 0.4 mg.kg^−1^ h^−1^ under inhalational anesthesia [22]. In the present study, rocuronium requirements for steady block PTC 1 – 2 in long duration (about 3 hours) were 113.2 ± 31.1 mg (0.6 mg.kg^−1^ h^−1^) in hypothermia and 115.8 ± 40.7 mg (0.7 mg.kg^−1^ h^−1^) in normothermia, which indicates that the requirement of rocuronium with continuous infusion was relatively reduced in hypothermia compared with normothermia (Table 1).

Greater degrees of change and heart rate fluctuations at 2 – 10 min were demonstrated after reversal with neostigmine–glycopyrrolate, compared with sugammadex [23]. In this study, there was a decrease in heart rate during 3 min after sugammadex reversal in both groups (Figure 2). The heart rate stability after sugammadex administration may allow hemodynamic stability and minimal cardiovascular adverse effects. The overall prevalence of adverse events after sugammadex was similar to placebo and neostigmine study groups [24]. Our study did not identify any PONV or other adverse effects following administration of sugammadex.

A peripheral skin temperature below 32°C with sustained and normal body temperature is associated with changes in both twitch tension and TOF ratio that may be a source of error when evaluating neuromuscular function [25]. The time course of action of NMB agents is prolonged by more than 50% in a cooled arm (skin temperature < 32°C) compared with a normothermic arm, which is especially unreliable in the PTC method [26]. So, the skin temperature over the monitored arm was more than 32°C which was maintained by wrapping the arm in cotton wool and forced-air warming in both groups in this study.

After the reversal of NMB by sugammadex, full recovery of the TOF ratio is possible when first twitch is still depressed. The TOF ratio as the only measurement for the adequate reversal of NMB by sugammadex may not always be reliable [27]. Although measurement of the TOF ratio is considered the standard in the present study, twitch height also has to be taken into account in the further study. The limitations of the current trial were no measurement of the plasma concentration of rocuronium, no evaluation of the twitch tension after the administration of sugammadex, and the limited sample size.

## Conclusions

Sugammadex can completely restore NMB from deep rocuronium-induced NMB in hypothermia. An additional 46 s was required for the time to TOF ratio 0.9 from a deep NMB in hypothermia patients. However, we propose that this prolonged recovery time is clinically acceptable.

## Authors’ information

HJL is an Assistant Professor in the Department of Anesthesiology and Pain Medicine, Hanyang University Hospital. KSK is a Professor in the Department of Anesthesiology and Pain Medicine, Hanyang University Hospital. JSJ is a Clinical Instructor in the Department of Anesthesiology and Pain Medicine, Samsung Seoul Hospital. KNK is a Clinical Fellow in the Department of Anesthesiology and Pain Medicine, Hanyang University Hospital. BCL is a Resident in the Department of Anesthesiology and Pain Medicine, Hanyang University Hospital.

## Abbreviations

BIS: Bispectral index
°C: Degrees Celsius
NMB: Neuromuscular block
PONV: Post-operative nausea and vomiting
PTC: Post-tetanic count
s: Seconds
TOF: Train-of-four.
